# Effects of Daily Mother–Infant Skin-to-Skin Contact on Breastfeeding Outcomes in the First Four Weeks and Maternal Postnatal Mental Health: A Quasi-Experimental Study

**DOI:** 10.3390/children13040570

**Published:** 2026-04-20

**Authors:** Chia-Wen Hung, Li-Min Wu

**Affiliations:** 1 Department of Nursing, Kaohsiung Chang Gung Memorial Hospital, Kaohsiung 833401, Taiwan; winne2280@gmail.com; 2 School of Nursing, Kaohsiung Medical University, Kaohsiung 807378, Taiwan; 3 Department of Medical Research, Kaohsiung Medical University Hospital, Kaohsiung 807377, Taiwan

**Keywords:** skin-to-skin contact, breastfeeding, lactation, postpartum period, maternal mental health, maternal–infant bonding, nursing intervention

## Abstract

**Highlights:**

**What are the main findings?**

- Daily skin-to-skin contact was associated with longer exclusive and continued breastfeeding duration.
- SSC was associated with improved lactation status during the postpartum period.

**What are the implication of the main findings?**

- Structured SSC can be implemented as a routine nursing intervention in postpartum care.
- Daily SSC may support breastfeeding sustainability and maternal recovery in real-world settings.

**Abstract:**

Background/Objectives: Skin-to-skin contact (SSC) between mother and infant is known to promote breastfeeding initiation and early bonding. However, evidence regarding the sustained effects of daily SSC during the postpartum period on breastfeeding outcomes and maternal mental health remains limited. This study aimed to evaluate the effects of structured daily SSC on breastfeeding outcomes, lactation status, and maternal postnatal mental health in a real-world clinical setting. Methods: A quasi-experimental design was used to compare mothers who performed daily SSC (SSC group) with those receiving care as usual (control group). Data were collected on postpartum Day 1, Day 3, Week 2, and Week 4. Primary outcomes included exclusive breastfeeding duration, continued breastfeeding duration, and lactation status. Multiple linear regression analyses adjusted for baseline breastfeeding intention and maternal age. Results: A total of 50 mother–infant dyads were included (SSC: *n* = 40; control: *n* = 10). The SSC group was associated with longer exclusive and continued breastfeeding durations and better lactation status (*p* < 0.05). Depressive symptoms did not differ significantly between groups, although both groups showed decreasing trends over time. After adjustment, daily SSC remained significantly associated with longer exclusive breastfeeding duration (adjusted *β* = 9.18 days, *p* = 0.034) and continued breastfeeding duration (adjusted *β* = 10.57 days, *p* = 0.001). Conclusions: Daily SSC is a simple and feasible intervention that may be associated with improved breastfeeding outcomes and lactation performance. Incorporating structured SSC into routine postpartum care may support breastfeeding sustainability and maternal recovery.

## 1. Background

Breastfeeding is widely recognized as the normative standard for infant feeding and is strongly recommended by the World Health Organization (WHO) and UNICEF as an essential component of early postnatal care [1,2]. Exclusive breastfeeding for the first six months of life is associated with reduced infant morbidity, improved immune protection, and enhanced maternal health outcomes [3]. Despite these recommendations, global breastfeeding rates remain suboptimal, highlighting the need for effective strategies to support breastfeeding sustainability [4].

Skin-to-skin contact (SSC), defined as placing the naked infant prone on the mother’s bare chest, has been incorporated into the Baby-Friendly Hospital Initiative as an evidence-based practice [2,5,6]. Early SSC improves breastfeeding initiation, increases breastfeeding duration, and stabilizes neonatal physiological parameters [5,7]. Previous evidence also suggests that SSC-related care practices may contribute to improved neonatal physiological stability and early adaptation [8]. Recent studies further support that SSC is associated with improved maternal and infant health outcomes, reinforcing its role in postpartum care [9].

The beneficial effects of SSC are partly mediated through activation of the oxytocinergic system, which promotes milk ejection, reduces maternal stress responses, and enhances maternal–infant bonding [10,11,12,13]. Oxytocin release during SSC is also associated with improved maternal psychological adaptation and attachment [14,15].

Although the short-term benefits of immediate SSC are well established, evidence regarding the sustained effects of structured daily SSC in healthy mother–infant dyads remains limited. Most existing studies focus on early breastfeeding initiation or neonatal physiological outcomes rather than longer-term breastfeeding sustainability and maternal psychological adaptation [7,16]. Randomized trials suggest that daily SSC may improve lactation and breastfeeding continuation; however, findings regarding its impact on postpartum depressive symptoms remain inconsistent [16].

Postpartum depressive symptoms and delayed lactogenesis are common concerns during the first month after childbirth and may contribute to early breastfeeding discontinuation [14,17]. From a neuroendocrine perspective, regular SSC may enhance oxytocin secretion, modulate stress responses, and support maternal emotional regulation [8,9,10].

Breastfeeding remains a critical public health issue in Taiwan. Although national initiatives such as the Baby-Friendly Hospital Initiative have improved breastfeeding initiation, the rate of exclusive breastfeeding declines during the early postpartum period [18]. Similar challenges have been reported in other Asian countries, where cultural practices, family dynamics, and postpartum care environments influence breastfeeding behaviors [3,4]. Although the benefits of early skin-to-skin contact (SSC) are well established, including improved breastfeeding initiation and maternal–infant bonding, recent evidence suggests that the effects of SSC on sustained breastfeeding outcomes remain variable across settings [19,20]. Therefore, further research is needed to evaluate the effectiveness of structured daily SSC in real-world clinical contexts, particularly regarding breastfeeding sustainability and maternal psychological adaptation. However, real-world clinical evidence examining both lactation function and maternal psychological recovery remains limited, particularly in healthy postpartum populations and in Asian contexts where sociocultural factors influence breastfeeding practices [21,22].

Therefore, this study aimed to examine the effects of structured daily SSC on lactation function, breastfeeding outcomes, and maternal postpartum mental health using a quasi-experimental design in a real-world clinical setting.

We hypothesized that daily skin-to-skin contact (SSC) would be associated with improved breastfeeding outcomes compared to routine postpartum care.

## 2. Materials and Methods

This quasi-experimental study evaluated the effects of daily mother–infant skin-to-skin contact (SSC) on lactation function, breastfeeding outcomes, and maternal postpartum mental health. Repeated measurements at multiple time points were performed to observe dynamic changes during postpartum recovery.

### 2.1. Setting and Participants

The study population consisted of healthy mother–infant dyads receiving routine postpartum care rather than high-risk or preterm populations. This study was conducted in the delivery room, postpartum ward, nursery, and postpartum care center of a medical center in southern Taiwan.

### 2.2. Inclusion Criteria

Mothers were eligible if they:

- Were aged ≥18 years;
- Had a singleton pregnancy;
- Delivered a clinically stable infant;
- Intended to breastfeed;
- Agreed to participate.

Infants were eligible if they:

- Were born at ≥36 weeks’ gestation;
- Had a birth weight ≥ 2000 g;
- Had no major congenital anomalies.

### 2.3. Exclusion Criteria

Participants were excluded if they had:

- Major obstetric complications;
- Infectious diseases;
- Medical contraindications to breastfeeding.

Eligible mothers were allocated to the SSC group or the control group according to their willingness to perform daily SSC, reflecting real-world clinical decision-making. Participants’ willingness to perform SSC was assessed during postpartum counseling by trained nursing staff using standardized verbal explanations of SSC procedures and benefits. Because allocation was based on maternal willingness, this approach may introduce selection bias, as mothers who chose to perform SSC may have higher baseline motivation for breastfeeding.

### 2.4. Intervention

Mothers in the SSC group performed skin-to-skin contact by placing the diaper-clad infant directly on the mother’s bare chest. SSC was performed 1–2 times daily, for at least 30 min per session, and continued for four weeks postpartum. Nursing staff provided standardized instruction on positioning, safety monitoring, thermal protection, and breastfeeding support. Nursing staff received standardized training prior to study implementation, including written protocols and practical demonstrations, to ensure consistent SSC guidance and safety monitoring across participants. SSC performance was documented. The control group received routine postpartum care and breastfeeding education but was not instructed to perform SSC at a fixed frequency.

### 2.5. Outcome Measures and Data Collection

This study collected maternal demographic characteristics, neonatal clinical data, and postpartum physiological and psychological outcomes.

### 2.6. Baseline Characteristics

Maternal variables included age, education level, employment status, parity, delivery mode, smoking and alcohol use, and breastfeeding intention.

Neonatal variables included sex, gestational age, birth weight, and Apgar scores at 1 and 5 min.

### 2.7. Maternal Psychological Status

Maternal depressive symptoms were assessed using the Edinburgh Postnatal Depression Scale (EPDS). The EPDS consists of 10 items scored from 0 to 3, with total scores ranging from 0 to 30. Higher scores indicate more severe depressive symptoms; a score ≥ 13 indicates probable depression. The EPDS is a validated screening tool widely used in postpartum populations.

### 2.8. Breastfeeding Outcomes Measures

Breastfeeding outcomes included:

1. Exclusive breastfeeding duration (days)

defined as the number of days the infant received only breast milk without formula supplementation.

2. Continued breastfeeding duration (days)

defined as the duration of any breastfeeding regardless of supplementation.

3. Lactation status

was assessed clinically and categorized into four levels: adequate, moderate, insufficient, or not measurable. The classification was based on structured clinical criteria, including milk production adequacy, infant feeding effectiveness (e.g., latch and feeding behavior), and the need for formula supplementation. These criteria are routinely applied in postpartum clinical practice and were evaluated by trained nursing staff.

No formal validated scale was used; however, a standardized clinical assessment based on routine postpartum care protocols was applied to ensure consistency in classification. To further enhance consistency, assessments were performed by designated nursing staff. As this variable represents an ordinal scale, non-parametric statistical methods were used for between-group comparisons.

The lactation status score was coded such that lower scores indicate better lactation performance.

4. Time to first breastfeeding (hours)

### 2.9. Postpartum Physical Recovery Indicators

Postpartum recovery indicators included:

- Uterine involution (days);
- Duration of lochia (days);
- Postpartum pain intensity assessed using a visual analog scale (VAS) ranging from 0 to 10.

### 2.10. Data Collection Procedure

All outcome measures were assessed by trained nurses at:

- Postpartum Day 1;
- Postpartum Day 3;
- Postpartum Week 2;
- Postpartum Week 4.

To support breastfeeding continuation, telephone follow-up was provided up to two months postpartum. Standardized quantitative assessments were conducted only within the first four weeks, as this period captures early breastfeeding establishment and maternal psychological adaptation, which are most dynamic during early postpartum recovery. Therefore, statistical analyses were based on data collected during this period. Longer-term follow-up is warranted in future studies.

Missing data were minimal, and complete-case analysis was performed. Adherence to SSC was monitored through nursing documentation records, and participant retention was supported through scheduled follow-up and telephone contact.

**Figure 1:**
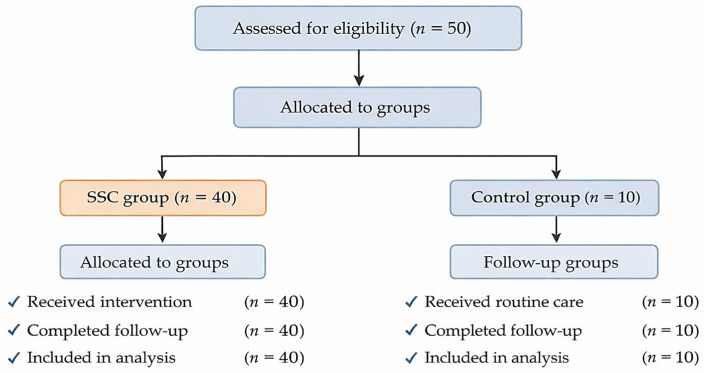
Participant flow diagram of recruitment, allocation, follow-up, and analysis.

A participant flow diagram was constructed to illustrate the processes of recruitment, allocation, follow-up, and analysis (Figure 1).

### 2.11. Statistical Analysis

Continuous variables were presented as medians and interquartile ranges (IQR), and categorical variables as frequencies and percentages. Due to the non-normal distribution of breastfeeding duration outcomes, data were summarized using medians and interquartile ranges, and non-parametric statistical methods were applied. This approach is consistent with standard reporting practices for skewed clinical data.

Between-group comparisons were performed using the Mann–Whitney U test for continuous variables and Fisher’s exact test for categorical variables. To evaluate the association between daily SSC and breastfeeding outcomes, multiple linear regression models were constructed. Exclusive breastfeeding duration and continued breastfeeding duration were entered as dependent variables. Because baseline breastfeeding intention differed significantly between groups, breastfeeding intention was included as the primary covariate. Maternal age and mode of delivery were also included to control for potential confounding effects. Adjusted regression coefficients (adjusted *β*), 95% confidence intervals (CI), and *p* values were reported. Multicollinearity among covariates was assessed using variance inflation factors (VIF), and all VIF values were below 2, indicating no significant multicollinearity. A two-tailed *p* value < 0.05 was considered statistically significant. All analyses were performed using SPSS version 22 (IBM Corp., Armonk, NY, USA). This study was reported in accordance with the STROBE guidelines.

### 2.12. Ethical Considerations

This study was approved by the Institutional Review Board (IRB) of Kaohsiung Chang Gung Memorial Hospital (IRB No. 202400470B0). Written informed consent was obtained from all participants prior to enrollment. The study was conducted in accordance with the principles of the Declaration of Helsinki.

## 3. Results

### 3.1. Participant Characteristics

A total of 50 mother–infant dyads were assessed for eligibility and included in the study, comprising 40 in the SSC group and 10 in the control group based on maternal willingness to perform SSC. All participants completed follow-up and were included in the final analysis. The participant flow is illustrated in Figure 1.

Baseline maternal and infant characteristics are presented in Table 1. There were no significant between-group differences in maternal age, education level, employment status, parity, delivery mode, or neonatal characteristics. However, baseline breastfeeding intention was significantly higher in the SSC group than in the control group (*p* = 0.02).

### 3.2. Breastfeeding Outcomes

Unadjusted comparisons demonstrated significant differences in breastfeeding outcomes between groups (Table 2). The median duration of exclusive breastfeeding was significantly longer in the SSC group. While mothers in the SSC group exclusively breastfed for a median of 6 days, formula supplementation was introduced early in the control group (*p* = 0.01). Notably, no infants in the control group achieved exclusive breastfeeding during the follow-up period. Although this distribution limits direct comparison between groups, it highlights a clinically meaningful contrast between routine care and the SSC intervention. Therefore, this outcome was retained to reflect real-world differences and should be interpreted with caution.

**Figure 2:**
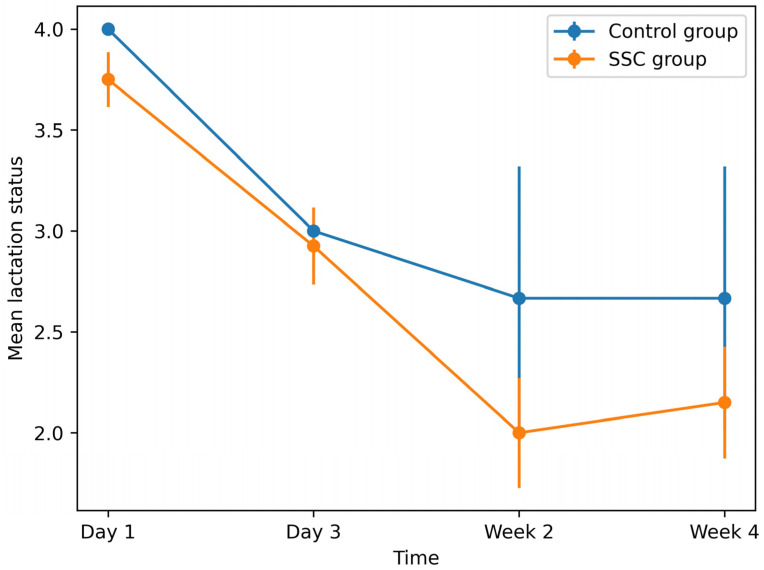
Trends in lactation status over time in the SSC and control groups. Error bars represent 95% confidence intervals. Lower scores indicate better lactation status. Blue line indicates the control group; orange line indicates the SSC group.

Continued breastfeeding duration was also significantly longer in the SSC group. Nearly all mothers in the SSC group maintained breastfeeding throughout the 4-week follow-up period, whereas the control group showed greater variability and shorter durations (*p* < 0.001). Lactation status differed significantly between groups on day one (*p* = 0.01), although this difference is unlikely to be attributable to the effects of sustained SSC and may reflect baseline variation between groups. Mothers in the SSC group demonstrated better lactation performance, as reflected by lower scores. Longitudinal trends demonstrated a steady improvement in lactation status in the SSC group, whereas improvement was less pronounced in the control group, although the difference was not statistically significant (Figure 2).

**Figure 3:**
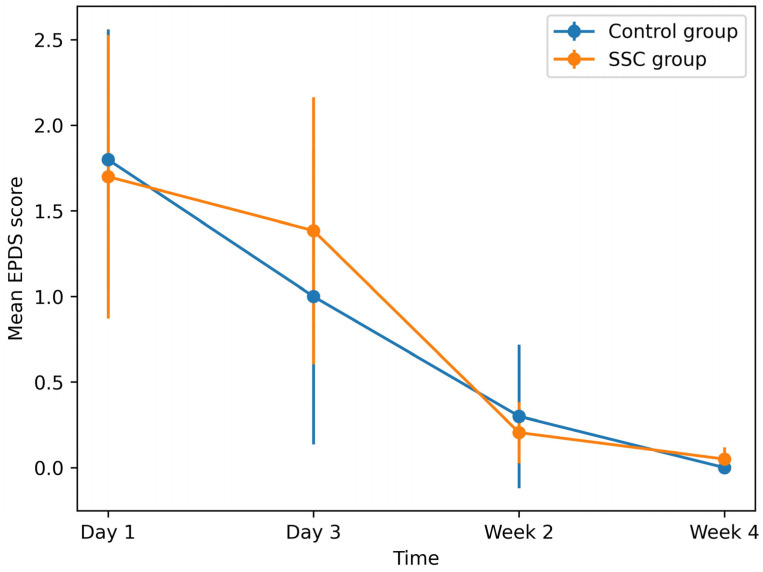
Trends in postpartum depressive symptoms (EPDS scores) over time in the SSC and control groups. Error bars represent 95% confidence intervals. Higher scores indicate more severe depressive symptoms. Blue line indicates the control group; orange line indicates the SSC group.

EPDS scores did not differ significantly between groups at any time point. However, depressive symptom scores decreased progressively across the postpartum period in both groups (Figure 3).

### 3.3. Postpartum Physical Recovery

No significant between-group differences were observed in postpartum recovery indicators, including time to first breastfeeding, uterine involution, duration of lochia, or postpartum pain scores.

### 3.4. Adjusted Analysis of Breastfeeding Outcomes

Multiple linear regression analyses adjusting for baseline breastfeeding intention and maternal age are presented in Table 3. After adjustment, daily SSC remained significantly associated with longer exclusive breastfeeding duration (adjusted *β* = 9.18 days, 95% CI 0.71–17.65, *p* = 0.034). Daily SSC was also significantly associated with longer continued breastfeeding duration (adjusted *β* = 10.57 days, 95% CI 4.88–16.26, *p* = 0.001). These findings suggest that the beneficial effects of SSC may extend beyond baseline breastfeeding intention; however, the results should be interpreted with caution.

## 4. Discussion

This study examined the effects of structured daily mother–infant skin-to-skin contact (SSC) on lactation function, breastfeeding outcomes, and maternal postpartum psychological recovery in a real-world clinical setting. The findings suggest that daily SSC was associated with longer exclusive and continued breastfeeding durations and improved lactation status compared with those receiving routine care; however, these findings should be interpreted with caution given the study design. These results are consistent with recent evidence indicating that SSC is associated with improved exclusive breastfeeding outcomes [23].

### 4.1. SSC and Breastfeeding Sustainability

Our findings reinforce extensive evidence that SSC is a critical facilitator of breastfeeding success. Early SSC has been shown to improve breastfeeding initiation and duration [5], while randomized trials demonstrate that daily SSC enhances breastfeeding continuation and milk production [7,16]. By extending SSC beyond the immediate postnatal period into structured daily practice, the present study provides real-world evidence that sustained SSC may be associated with breastfeeding sustainability. Nearly all mothers in the SSC group maintained breastfeeding throughout the 4-week follow-up period, whereas the control group exhibited greater variability and earlier discontinuation. Early cessation of breastfeeding remains a major global public health challenge despite strong public health recommendations [3,4]. Breastfeeding continuation is influenced not only by physiological readiness but also by maternal confidence and early feeding experiences [24]. According to breastfeeding self-efficacy theory, maternal confidence plays a central role in breastfeeding persistence [25]. Our findings extend existing evidence by demonstrating that structured daily SSC is associated with breastfeeding sustainability in healthy postpartum populations. While early SSC immediately after birth has been widely promoted, maintaining SSC as a daily practice during the postpartum period appears to be associated with additional benefits in sustaining breastfeeding behaviors. In this study, mothers who engaged in daily SSC were more likely to maintain breastfeeding throughout the follow-up period, suggesting that repeated mother–infant contact may be associated with maternal confidence and feeding interactions in real-world settings.

### 4.2. Neuroendocrine Mechanisms and Lactation Physiology

It should be noted that differences observed on day one are unlikely to be attributable to the effects of sustained SSC and may reflect baseline variation between groups. The improvement in lactation status observed among SSC participants after the initial postpartum period is biologically plausible and may be explained by neuroendocrine mechanisms activated during mother–infant skin-to-skin contact. SSC stimulates oxytocin release, which plays a central role in milk ejection and promotes maternal relaxation and bonding [10,11,12]. In addition, oxytocin release during breastfeeding is associated with increased prolactin secretion, enhanced milk production, reduced cortisol levels, and improved maternal emotional regulation [26]. These hormonal effects create a physiological environment conducive to lactogenesis and effective milk transfer. Beyond endocrine pathways, SSC promotes autonomic regulation and physiological stability in both mothers and infants [15]. This neuroendocrine synchronization may enhance maternal responsiveness to infant feeding cues and improve breastfeeding effectiveness. Furthermore, SSC has been shown to increase maternal oxytocin levels and enhance early parent–infant bonding, further supporting effective feeding interactions [11]. SSC also supports parent–infant synchrony, characterized by coordinated behavioral and physiological interactions that promote infant neurodevelopment and emotional regulation [27]. Such synchronous interactions strengthen maternal sensitivity and bonding, further supporting effective feeding. Collectively, these neuroendocrine and behavioral mechanisms provide a plausible physiological basis for the improved lactation performance observed in the SSC group.

### 4.3. Maternal Psychological Adaptation

Although depressive symptom scores did not differ significantly between groups, these findings should be interpreted with caution. The relatively small and imbalanced sample size may have limited statistical power, increasing the risk of a Type II error. Therefore, the absence of significant differences does not necessarily indicate a lack of effect of SSC on maternal mental health. Both groups demonstrated progressive improvement during the postpartum period. SSC may contribute to maternal emotional adaptation through enhanced bonding, stress modulation, and oxytocin-mediated calming effects [14,21]. Previous studies indicate that SSC reduces maternal stress and promotes psychological well-being [14]; however, evidence regarding postpartum depressive symptoms remains inconsistent [16], which is consistent with the findings of this study. In addition, the absence of significant group differences may also be explained by low baseline depressive symptom levels and comprehensive postpartum support provided to all participants. Psychological benefits may emerge over longer follow-up periods, as maternal emotional adjustment continues beyond the first postpartum month.

### 4.4. Postpartum Recovery Outcomes

No significant differences were observed in postpartum recovery indicators, including uterine involution, lochia duration, and pain scores. Postpartum physiological recovery is influenced by multiple factors, including delivery mode, maternal health status, and postpartum care practices. Previous research has similarly reported limited effects of SSC on physical recovery outcomes despite psychological and bonding benefits [17]. These findings suggest that SSC primarily influences neurobehavioral and lactation-related processes rather than physiological healing parameters.

### 4.5. Cultural and Contextual Considerations

Breastfeeding behaviors are shaped by sociocultural norms, family support, and healthcare environments. In Asian contexts, cultural practices and postpartum care traditions, such as family involvement and confinement practices, may influence maternal feeding decisions and engagement in breastfeeding-related behaviors [16]. These sociocultural factors may also affect the implementation and sustainability of SSC in routine care. Our findings are consistent with previous research suggesting that breastfeeding outcomes are influenced not only by physiological factors but also by cultural and environmental contexts [3,4]. In settings where family support and postpartum care practices are aligned with breastfeeding promotion, SSC may be more effectively integrated into daily routines. Furthermore, variability in SSC effectiveness across studies has been attributed to differences in implementation context and maternal characteristics [19]. The present study extends this perspective by demonstrating that structured daily SSC can be successfully implemented within a real-world clinical setting in Taiwan, suggesting that culturally adapted SSC interventions may be associated with supporting breastfeeding sustainability. Integrating SSC into routine postpartum care aligns with global recommendations and may help address sociocultural barriers to breastfeeding in diverse healthcare settings [28].

### 4.6. Clinical Implications for Nursing Practice

Daily SSC represents a simple, low-cost, and feasible nursing intervention that can be integrated into routine postpartum care. Unlike single-time SSC immediately after birth, structured daily SSC supports ongoing lactation stimulation, maternal–infant interaction, and maternal confidence in caregiving. From a nursing perspective, SSC can be implemented across postpartum wards, baby-friendly hospital initiatives, and community follow-up programs. Education and support from nursing staff are essential to ensure safe positioning, maternal comfort, and sustained practice. Given its non-invasive nature and minimal resource requirements, SSC is suitable for widespread adoption in diverse healthcare settings.

### 4.7. Strengths and Limitations

This study has several strengths. First, it was conducted in a real-world clinical setting, enhancing ecological validity. Second, repeated measurements enabled evaluation of dynamic changes in lactation and psychological outcomes. Third, the intervention is simple and reproducible in routine care.

Several limitations should be acknowledged. The non-randomized design and self-selection into groups may introduce selection bias, and residual confounding may persist despite adjusted analyses. Mothers who chose to perform SSC may have had higher intrinsic motivation for breastfeeding, which could influence outcomes. Although baseline breastfeeding intention was adjusted for in the analysis, residual confounding cannot be fully eliminated. In addition, the imbalance in group sizes may limit statistical power and generalizability, and may affect the precision of the estimates. These methodological constraints should be considered when interpreting the findings. The relatively small control group may further limit statistical power, particularly in detecting differences in maternal psychological outcomes. Additionally, psychological outcomes were assessed within four weeks postpartum; longer follow-up may be required to evaluate sustained mental health benefits.

### 4.8. Implications for Future Research

In addition, the imbalance in group sizes and the self-selection of participants into the SSC group may introduce selection bias and limit generalizability. Although key confounders such as breastfeeding intention were adjusted for in the analysis, residual confounding cannot be excluded. Future randomized studies with larger samples are warranted to confirm the causal effects of daily SSC on breastfeeding sustainability. Extended follow-up periods may clarify long-term psychological outcomes and maternal–infant bonding trajectories. Research across diverse cultural contexts is also needed to enhance generalizability and inform culturally responsive breastfeeding promotion strategies.

These findings support the integration of SSC into breastfeeding promotion strategies worldwide.

### 4.9. Clinical Implications

Given that participation in SSC was based on maternal willingness, implementation in clinical practice should focus on promoting SSC through education, encouragement, and supportive care environments rather than mandatory protocols. Providing structured guidance and supportive care environments may enhance maternal engagement in SSC and improve breastfeeding outcomes in routine postpartum care.

## 5. Conclusions

Structured daily mother–infant skin-to-skin contact may be associated with improved breastfeeding outcomes and lactation status during the early postpartum period. These findings suggest that SSC may serve as a feasible and supportive nursing intervention to promote breastfeeding sustainability in routine clinical practice. Although no significant differences were observed in maternal psychological outcomes, SSC may still contribute to maternal emotional adaptation during postpartum recovery. However, given the quasi-experimental design and sample limitations, the results should be interpreted with caution, and further studies with larger samples and longer follow-up are needed to confirm these findings.

## Figures and Tables

**Table 1:** Baseline characteristics of mothers and infants.

Variable	SSC (*n* = 40)	Control (*n* = 10)	*p* Value
**Maternal characteristics**			
Age, years, median (IQR)	34.0 (31.3–40.0)	34.5 (29.5–36.0)	0.45
Education ≥ college, *n* (%)	30 (75.0)	9 (90.0)	0.79
Employment, *n* (%)	33 (82.5)	9 (90.0)	0.09
Smoking, *n* (%)	0 (0.0)	0 (0.0)	–
Alcohol use, *n* (%)	0 (0.0)	0 (0.0)	–
Vaginal delivery, *n* (%)	20 (50.0)	4 (40.0)	0.73
Parity (G), median (IQR)	2.0 (1–3)	2.0 (1–4)	1.00
Breastfeeding intention, *n* (%)	37 (92.5)	3 (30.0)	0.02
**Infant characteristics**			
Gestational age, weeks	272 (266–276.5)	272 (268.8–284.0)	0.23
Birth weight, g	3050 (2760–3285)	3140 (2940–3285)	0.48
Apgar score (1 min)	9 (9–9)	9 (9–9)	–
Apgar score (5 min)	10 (10–10)	10 (10–10)	–

Note: Values are presented as median (IQR) or *n* (%). Bold text indicates category headers.

**Table 2:** Comparison of breastfeeding and postpartum outcomes between groups.

Outcome	SSC Group	Control Group	*p* Value
Breastfeeding outcomes			
Exclusive breastfeeding duration (days)	6 (0–28)	0 (0–0)	0.01
Continued breastfeeding duration (days)	28 (28–28)	20 (0–28)	<0.001
Lactation outcomes			
Lactation status score, Day 1	4.0 (3.3–4.0)	4.0 (4.0–4.0)	0.01
Lactation status score, Day 3	3.0 (3.0–3.0)	3.0 (3.0–3.0)	0.237
Lactation status score, Week 2	2.0 (1.0–3.0)	3.0 (2.5–3.0)	0.083
Lactation status score, Week 4	2.0 (1.0–3.0)	3.0 (2.5–3.0)	0.091
Maternal outcomes			
EPDS score, Day 1	0 (0–3.8)	2 (0.8–2.3)	0.90
First breastfeeding time (hours)	7 (3–16)	7.5 (4–18)	0.57
Uterine involution (days)	8 (7–10)	8.5 (7–10)	0.77
Lochia duration (days)	26 (25–27.8)	26.5 (25–28)	0.88
Pain score (VAS), Day 1	3 (3–4)	3 (3–4)	0.68

Note: Values are presented as median (interquartile range).

**Table 3:** Adjusted multiple linear regression models for breastfeeding outcomes.

Outcome	Variable	Adjusted *β*	95% CI	*p* Value
Exclusive breastfeeding (days)	SSC (vs control)	9.18	0.71–17.65	0.034
	Breastfeeding intention	1.31	−6.57–9.19	0.740
	Maternal age	−0.08	−0.72–0.55	0.790
Continued breastfeeding (days)	SSC (vs control)	10.57	4.88–16.26	0.001
	Breastfeeding intention	−4.80	−10.09–0.49	0.075
	Maternal age	−0.04	−0.47–0.39	0.854

Note: Models adjusted for breastfeeding intention and maternal age. *β* represents adjusted regression coefficient; CI = confidence interval. Model diagnostics indicated acceptable model fit.

## Data Availability

The data presented in this study are available on request from the corresponding author due to privacy and ethical restrictions.

## Abbreviations

SSC	Skin-to-skin contact
EPDS	Edinburgh Postnatal Depression Scale
CI	Confidence interval
